# Morphological variables associated with ruptured basilar tip aneurysms

**DOI:** 10.1038/s41598-021-81364-8

**Published:** 2021-01-28

**Authors:** Jian Zhang, Anil Can, Pui Man Rosalind Lai, Srinivasan Mukundan, Victor M. Castro, Dmitriy Dligach, Sean Finan, Vivian S. Gainer, Nancy A. Shadick, Guergana Savova, Shawn N. Murphy, Tianxi Cai, Scott T. Weiss, Rose Du

**Affiliations:** 1 Department of Neurosurgery, Brigham and Women’s Hospital, 75 Francis Street, Boston, MA 02115 USA; 2 Department of Neurosurgery and Brain and Nerve Research Laboratory, The First Affiliated Hospital of Soochow University, Suzhou, Jiangsu Province China; 3 Department of Neurosurgery, Amsterdam University Medical Centers, Amsterdam, The Netherlands; 4 Department of Radiology, Brigham and Women’s Hospital, Boston, MA USA; 5 Research Information Systems and Computing, Massachusetts General Brigham, Boston, MA USA; 6 Boston Children’s Hospital Informatics Program, Boston, MA USA; 7 Department of Computer Science, Loyola University, Chicago, IL USA; 8 Division of Rheumatology, Immunology and Allergy, Brigham and Women’s Hospital, Boston, MA USA; 9 Department of Neurology, Massachusetts General Hospital, Boston, MA USA; 10 Harvard T.H. Chan School of Public Health, Boston, MA USA; 11 Channing Division of Network Medicine, Brigham and Women’s Hospital, Boston, MA USA

**Keywords:** Cerebrovascular disorders, Stroke

## Abstract

Morphological factors of intracranial aneurysms and the surrounding vasculature could affect aneurysm rupture risk in a location specific manner. Our goal was to identify image-based morphological parameters that correlated with ruptured basilar tip aneurysms. Three-dimensional morphological parameters obtained from CT-angiography (CTA) or digital subtraction angiography (DSA) from 200 patients with basilar tip aneurysms diagnosed at the Brigham and Women’s Hospital and Massachusetts General Hospital between 1990 and 2016 were evaluated. We examined aneurysm wall irregularity, the presence of daughter domes, hypoplastic, aplastic or fetal PCoAs, vertebral dominance, maximum height, perpendicular height, width, neck diameter, aspect and size ratio, height/width ratio, and diameters and angles of surrounding parent and daughter vessels. Univariable and multivariable statistical analyses were performed to determine statistical significance. In multivariable analysis, presence of a daughter dome, aspect ratio, and larger flow angle were significantly associated with rupture status. We also introduced two new variables, diameter size ratio and parent-daughter angle ratio, which were both significantly inversely associated with ruptured basilar tip aneurysms. Notably, multivariable analyses also showed that larger diameter size ratio was associated with higher Hunt-Hess score while smaller flow angle was associated with higher Fisher grade. These easily measurable parameters, including a new parameter that is unlikely to be affected by the formation of the aneurysm, could aid in screening strategies in high-risk patients with basilar tip aneurysms. One should note, however, that the changes in parameters related to aneurysm morphology may be secondary to aneurysm rupture rather than causal.

## Introduction

Intracranial aneurysm formation and rupture are believed to be multifactorial, involving genetic, environmental, and geometric risk factors^1–3^. Since hemodynamic stress—which is thought to play an important role in rupture by triggering focal degenerative mechanisms at the vessel wall—is affected by aneurysm anatomy and the geometry of the surrounding vasculature, investigating the effects of these parameters in a location specific manner could aid in understanding the risk of aneurysm rupture^4–13^. We aimed to study the features associated with rupture in a large sample of 200 basilar tip aneurysms that were examined using morphological and clinical variables. Since unruptured basilar tip aneurysms account for only 3% of all intracranial aneurysms, our study is unique in the large number of aneurysms, and the detailed inclusion of new variables that involve the surrounding vasculature which are not intrinsic to aneurysm morphology and therefore unlikely to be affected by the formation of the aneurysm^1^.

## Methods

### Patient selection

Patients diagnosed with an intracranial aneurysm at the Brigham and Women’s Hospital (BWH) and Massachusetts General Hospital (MGH) from 1990–2016 were identified using natural language processing (NLP) in conjunction with manual medical record review from the Partners Healthcare Research Patients Data Registry (RPDR)^14^. This registry includes 4.2 million patients who have received care from BWH and MGH. 5,589 patients with potential aneurysms were identified from the RPDR using a machine learning algorithm based on both codified and NLP data^14^. Of these patients, 727 patients were also seen on clinical presentation from 2007–2013 with prospectively collected data^14^. An additional 474 patients with prospectively collected data who were seen on clinical presentation from 2013–2016, were also included, resulting in a total of 6,063 patients^14^. By manually reviewing (AC and RD) the medical records of all 6,063 patients, 4,701 patients with definite saccular aneurysms were identified^14^. Two-hundred patients with basilar artery aneurysms had available imaging of sufficient quality which were obtained using the mi2b2 open-source software to comply with research privacy requirements. Non-saccular (fusiform) aneurysms or aneurysms associated with arteriovenous malformations were excluded from this study. Demographic and clinical information, including Hunt-Hess score, tobacco and alcohol use, history of hypertension, and family history of intracranial aneurysms and subarachnoid hemorrhage, was retrieved from medical records. For patients with ruptured aneurysms, images were reviewed for Fisher grades. Patients who had no objective signs of subarachnoid hemorrhage by CT or lumbar puncture were considered unruptured. This study was approved by the Partners Institutional Review Board and considered minimal risk. Informed consent was therefore waived. All procedures performed were in accordance with the ethical standards of the institutional review board and with the 1964 Helsinki declaration and its later amendments or comparable ethical standards.

### Reconstruction of 3D models

Using preoperative CTA via the Vitrea Advanced Visualization software (version 6.9.68.1, Vital Images, Minnetonka, MN), three-dimensional (3D) models of aneurysms and their surrounding vasculature were generated^15^. The software creates a spatial reconstruction of the vasculature from axial CTA images in the DICOM (Digital Images and Communication in Medicine) format. DSA studies with 3D reconstructions were evaluated directly when a CTA of sufficient quality was unavailable^16^. We manually measured lengths and angles. In order to ensure accurate measurements, windowing for the 3D reconstructions were validated against the multiplanar reconstructions^16^. Measurements were performed by an attending neurosurgeon (JZ) and verified by a second (RD) when needed.

### Definition of morphological parameters

**Figure 1:**
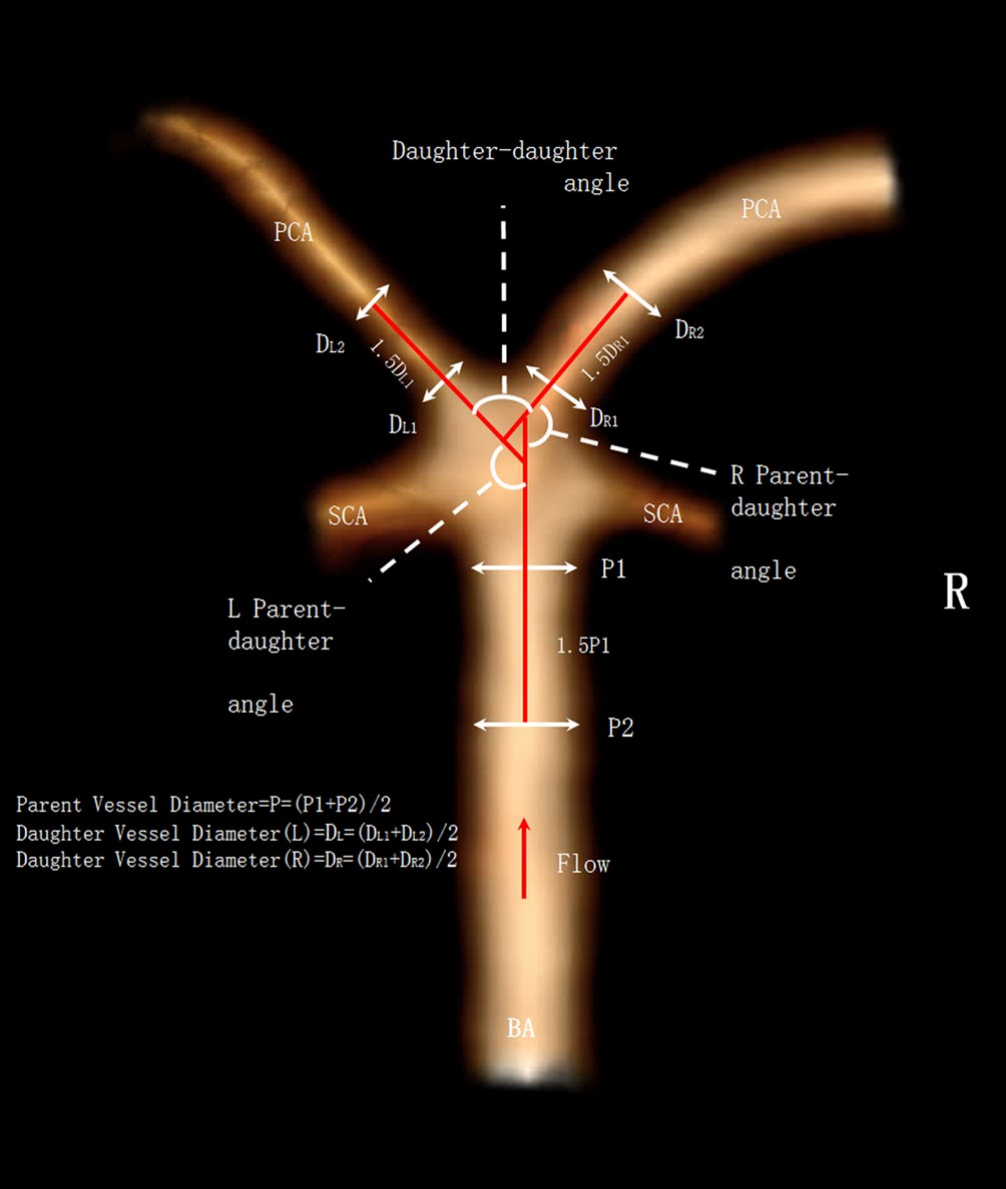
Illustrations of morphological parameters. Image was partially created by Vitrea Advanced Visualization software (version 6.9.68.1).

**Figure 2:**
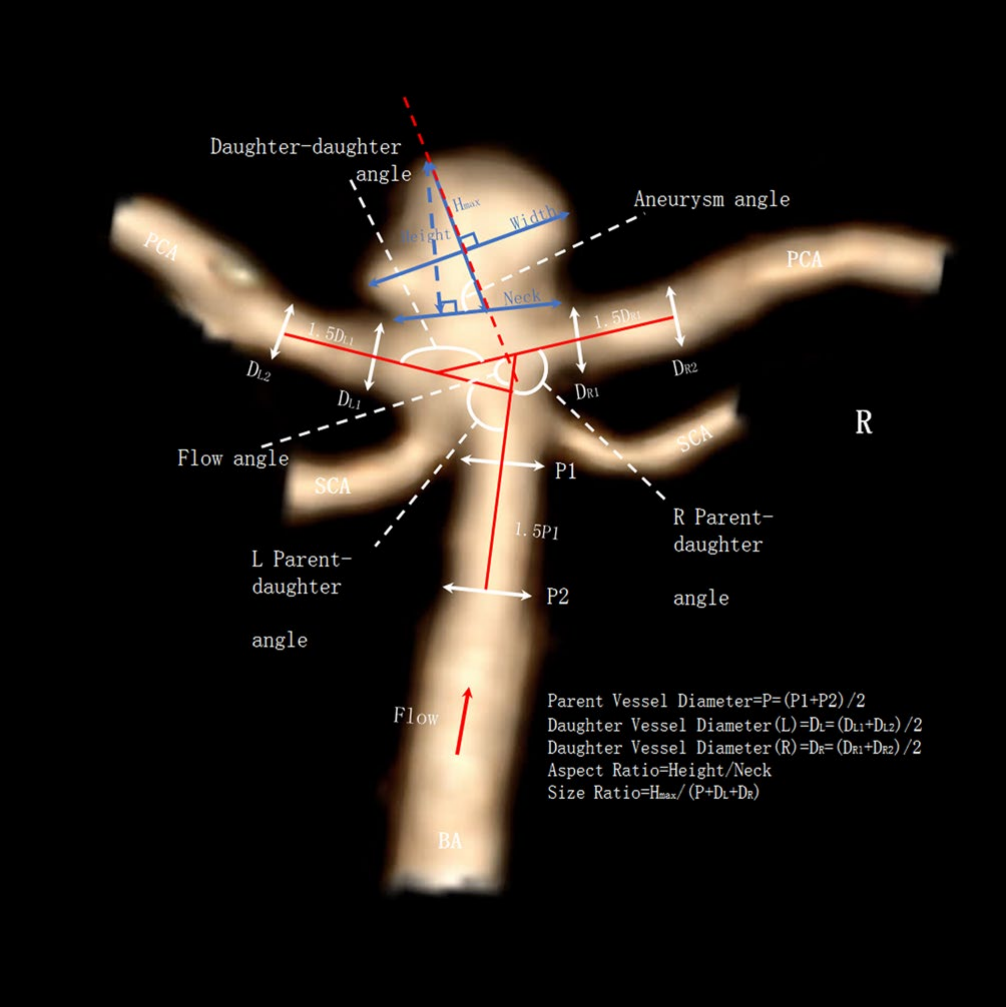
Illustrations of morphological parameters. Image was partially created by Vitrea Advanced Visualization software (version 6.9.68.1).

Both aneurysm related variables and measurements of the surrounding vasculature were used in our study^16^, and are described briefly below (Figs. 1, 2). Basilar tip aneurysms were categorized as smooth or irregular (non-smooth wall), and with or without daughter domes. If hypoplastic or aplastic posterior communicating arteries (PCoAs) and/or fetal PCoAs were present, the number of vessels with this anatomical variation was noted (e.g. single or double). A PCoA was considered hypoplastic if its diameter was less than half of the contralateral PCoA. A PCoA was considered aplastic if it was not visible on CTA. Vertebral artery dominance was defined as the presence of unequal vertebral artery diameters. Maximum aneurysm height was defined as the length between the center of the aneurysm neck and the greatest distance to the dome, whereas maximum perpendicular height was the largest perpendicular distance from the neck of the aneurysm to the dome of the aneurysm. We also measured the neck diameter, the width of the aneurysm (maximal diameter perpendicular to maximum height line), and the aspect ratio (AR) which was calculated as the ratio of the maximum perpendicular height of the aneurysm to the average neck diameter of the aneurysm. Height/width ratio was defined as the ratio of maximum perpendicular height to width. Size ratio was calculated by dividing the maximum height by the mean vessel diameter of all branches (parent and daughter arteries) associated with the aneurysm. Vessel diameters were measured by averaging the diameter of the cross-section of a vessel (D) just proximal to the neck of the aneurysm and the diameter of the cross-section at 1.5 times D from the neck of the aneurysm. Average diameters of the parent artery, larger daughter branch and the smaller daughter branches were calculated in this manner. The diameter size ratio was defined as the parent artery diameter divided by the sum of the diameters of both daughter branches, and the daughter diameter ratio was defined as the larger daughter artery diameter divided by the smaller daughter artery diameter. Daughter-daughter angle was defined as the angle formed between the daughter vessels, parent-daughter angle was the angle between the parent vessel and the daughter vessel, and the flow angle was the angle between the maximum height of the aneurysm and the parent vessel.

### Statistical analysis

Differences in baseline characteristics between the ruptured and unruptured groups were calculated using the *t-*test for continuous variables and the Pearson's chi-square test for categorical variables. Univariable and multivariable logistic regression models were used to test for effects of different morphological parameters on rupture status, with a backward elimination procedure to identify significant confounders. We used cut-off values of 0.1 in order to select the initial set of variables to be included in the initial multivariable model for backward elimination. Firth’s bias reduction was used to address the issue of complete separation^17^. Adjusted odds ratios (OR) with 95% confidence intervals (CIs) were calculated and p < 0.05 was considered significant. All statistical analyses were performed using the Stata statistical software package (version 14, StataCorp. College Station, TX) or R^18^ (version 4.0.2).

## Results

Two-hundred patients with basilar tip aneurysms were included in this study. Table 1 shows the demographic data and clinical risk factors of patient with ruptured and unruptured basilar tip aneurysms. The mean patient age was 57.0 (11.0 SD), and 75.5% of patients were female. Patients with ruptured aneurysms were younger, less frequently female, more frequently alcohol and tobacco users, and were less likely to have hypertension, although none of these differences were statistically significant (Table 1).

**Table 1:** Demographic data and clinical risk factors of patients with ruptured and unruptured basilar tip aneurysms (N = 200).

Variables	All patients N = 200	Patients with ruptured aneurysms N = 73	Patients with unruptured aneurysms N = 127	*P* value
Age (SD)	57.0 (11.0)	56.6 (12.0)	57.1 (10.5)	0.75
Female (%)	151 (75.5)	53 (72.6)	98 (77.2)	0.58
Alcohol use (current) (%)	94 (47.0)	40 (54.8)	54 (42.5)	0.13
Tobacco use (current) (%)	80 (40.0)	31 (42.5)	49 (38.6)	0.70
Hypertension (%)	107 (53.5)	37 (50.7)	70 (55.1)	0.65
Family history of SAH (%)	23 (11.5)	9 (12.3)	14 (11.0)	0.96
Family history of aneurysms	40 (20.0)	14 (19.2)	26 (20.5)	0.97

SAH = subarachnoid hemorrhage.

We then examined the predefined aneurysm characteristics of the ruptured and unruptured basilar tip aneurysms (Table 2). Ruptured aneurysms were more frequently irregular (53% vs 20%) with daughter domes (63% vs 20%). In addition, ruptured aneurysms had a greater aspect ratio (1.5 vs 1.2), height/width ratio (1.01 vs 0.92), and flow angle (142 vs 131). In contrast, ruptured aneurysms had a smaller diameter size ratio (0.69 vs 0.73), and parent-daughter angle ratio (1.2 vs 1.3). There was no significant difference in posterior projection, maximum height, perpendicular height, diameter neck, aneurysm width, hypoplastic, aplastic or fetal PCoA, vertebral dominance, size ratio, daughter diameter ratio, parent artery diameter, and daughter-daughter angles between the ruptured and unruptured groups.

**Table 2:** Aneurysm characteristics stratified by rupture status of basilar artery aneurysms (N = 200).

Variables	All N = 200	Ruptured N = 73	Unruptured N = 127	*P* value
Irregular (%)	65 (32.5)	39 (53.4)	26 (20.4)	< 0.01
Daughter dome (%)	71 (35.5)	46 (63.0)	25 (19.7)	< 0.01
Posterior projection (%)	54 (27.0)	15 (20.5)	39 (30.7)	0.16
Hypoplastic PCoA (%)				
No	119 (59.5)	46 (63.0)	73 (57.5)	0.62
Unilateral	43 (21.5)	13 (17.8)	30 (23.6)	
Bilateral	38 (19.0)	14 (19.2)	24 (18.9)	
Aplastic PCoA (%)				
No	189 (94.5)	70 (95.9)	119 (93.7)	0.43
Unilateral	7 (3.5)	1 (1.4)	6 (4.7)	
Bilateral	4 (2.0)	2 (2.7)	2 (1.6)	
Fetal PCoA (%)				
No	183 (91.5)	69 (94.5)	114 (89.8)	0.22
Unilateral	12 (6.0)	4 (5.5)	8 (6.3)	
Bilateral	5 (2.5)	0 (0.0)	5 (3.9)	
Vertebral dominance (%)	41 (20.5)	18 (24.7)	23 (18.1)	0.36
Maximum height in mm (SD)	6.69 (3.90)	7.20 (3.67)	6.4 (4.01)	0.15
Perpendicular height in mm (SD)	6.08 (3.77)	6.50 (3.65)	5.84 (3.83)	0.23
Diameter neck in mm (SD)	4.66 (2.20)	4.34 (1.38)	4.84 (2.55)	0.07
Width aneurysm in mm (SD)	6.52 (4.05)	6.55 (3.25)	6.51 (4.32)	0.94
Aspect ratio (SD)	1.30 (0.59)	1.49 (0.66)	1.20 (0.51)	< 0.01
Height/width ratio	0.95 (0.29)	1.01 (0.33)	0.92 (0.27)	0.03
Daughter diameter ratio (larger/smaller) (SD)	1.18 (0.22)	1.14 (0.16)	1.20 (0.24)	0.06
Parent artery (basilar artery) diameter in mm (SD)	2.50 (0.47)	2.44 (0.45)	2.53 (0.48)	0.22
Diameter size ratio (Parent/(D1 + D2))	0.71 (0.11)	0.69 (0.13)	0.73 (0.10)	0.02
Size ratio (SD)	1.16 (0.76)	1.25 (0.74)	1.10 (0.76)	0.16
Daughter-daughter angle in degrees (SD)	139.9 (21.7)	142.0 (20.1)	138.6 (22.6)	0.27
Parent-daughter angle ratio (SD)	1.29 (0.29)	1.21 (0.22)	1.33 (0.31)	< 0.01
Flow angle in degrees (SD)	134.9 (19.6)	142.3 (18.3)	130.8 (19.2)	< 0.01

PCoA = posterior communicating artery.

Table 3 shows the results of the univariable and multivariable analyses for rupture status of the basilar tip aneurysms. In the univariable analysis, irregularity (OR 4.46, 95% CI 2.39–8.57), presence of a daughter dome (OR 6.95, 95% CI 3.69–13.47), larger flow angle (OR 1.03, 95% CI 1.02–1.05), larger aspect ratio (OR 2.40, 95% CI 1.42–4.29) and larger height/width ratio (OR 3.16, 95% CI 1.16–9.45) were significantly associated with aneurysm rupture. In addition, parent-daughter angle ratio (OR 0.18, 95% CI 0.05–0.56) and diameter size ratio (OR 0.02, 95% CI 0.001–0.41) were significantly and inversely associated with ruptured status. In multivariable analysis, presence of a daughter dome (OR 5.53, 95% CI 2.67–11.45), larger aspect ratio (OR 2.39, 95% CI 1.18–4.86), and larger flow angle (OR 1.04, 95% CI 1.02–1.05) were significantly associated with rupture status. In contrast, diameter size ratio (OR 0.01, 95% CI 0.00–0.35) and parent-daughter angle ratio (OR 0.08, 95% CI 0.02–0.38) were significantly inversely associated with ruptured basilar tip aneurysms. When stratified according to Hunt-Hess score and Fisher grade among ruptured aneurysms, higher diameter size ratio (β 6.19, 95% CI 2.42–9.95) was associated with higher Hunt-Hess score and lower flow angle (β − 0.03, 95% CI − 0.06—− 0.001) was associated with higher Fisher grade in the multivariable analyses (Table 4). Finally, when stratified by presence of multiple aneurysms, none of the morphological variables was significant.

**Table 3:** Univariable and multivariable logistic regression for rupture status (N = 200).

Variables	Univariable		Multivariable	
	OR (95% CI)	*P* value	OR (95% CI)	*P* value
Daughter dome	6.95 (3.69–13.47)	< 0.01	5.53 (2.67–11.45)	< 0.01
Aspect ratio	2.40 (1.42–4.29)	< 0.01	2.39 (1.18–4.86)	0.02
Flow angle	1.03 (1.02–1.05)	< 0.01	1.04 (1.02–1.05)	< 0.01
Parent-daughter angle ratio	0.18 (0.05–0.56)	< 0.01	0.08 (0.02–0.38)	< 0.01
Diameter size ratio (Parent/(D1 + D2))	0.02 (0.001–0.41)	0.01	0.01 (0.00–0.35)	0.01
Irregular	4.46 (2.39–8.47)	< 0.01	–	–
Posterior projection	0.58 (0.29–1.14)	0.12	–	–
Hypoplastic PCoA				
Unilateral (vs. no)	0.69 (0.32–1.43)	0.33	–	–
Bilateral (vs. no)	0.93 (0.43–1.95)	0.84	–	–
Aplastic PCoA				
Unilateral (vs. no)	0.28 (0.01–1.71)	0.25	–	–
Bilateral (vs. no)	1.70 (0.20–1.42)	0.60	–	–
Fetal PCoA				
Unilateral (vs. no)	0.87 (0.24–2.74)	0.82	–	–
Bilateral (vs. no)	0.15 (0.001–1.35)	0.10	–	–
Vertebral dominance	1.48 (0.73–2.97)	0.27	–	–
Maximum height in mm	1.05 (0.98–1.14)	0.16	–	–
Perpendicular height in mm	1.05 (0.97–1.13)	0.23	–	–
Diameter neck in mm	0.89 (0.75–1.02)	0.13	–	–
Width aneurysm in mm	1.00 (0.93–1.08)	0.95	–	–
Height/width ratio	3.16 (1.16–9.45)	0.03	–	–
Daughter diameter ratio (larger/smaller)	0.25 (0.04–1.11)	0.09	–	–
Parent artery (basilar artery) diameter	1.30 (0.89–1.91)	0.17	–	–
Size ratio	1.31 (0.90–1.91)	0.16	–	–
Daughter-daughter angle	1.01 (0.99–1.02)	0.29	–	–

PCoA = posterior communicating artery.

**Table 4:** Univariable and multivariable logistic regression for Hunt-Hess score and Fisher grade in ruptured basilar artery aneurysms (N = 71*).

Variables	Hunt-HESS score				Fisher grade			
	Univariable		Multivariable		Univariable		Multivariable	
	Coef (95% CI)	*P* value	Coef (95% CI)	*P* value	Coef (95% CI)	*P* value	Coef (95% CI)	*P* value
Daughter dome	− 0.02 (− 0.88 to 0.83)	0.96	–	–	− 1.05 (− 2.03 to − 0.06)	0.04	− 0.95 (− 1.96 to 0.06)	0.06
Aspect ratio	0.18 (− 0.47 to 0.84)	0.59	–	–	0.60 (− 0.25–1.44)	0.17	-	-
Flow angle	− 0.01 (− 0.04 to 0.01)	0.37	-	–	− 0.03 (− 0.06 to − 0.004)	0.03	− 0.03 (− 0.06 to − 0.001)	0.04
Parent-daughter angle ratio	2.05 (0.08 to 4.03)	0.04	1.99 (− 0.08 to 4.06)	0.06	0.16 (− 1.94 to 2.27)	0.88	–	–
Diameter size ratio (Parent/(D1 + D2))	6.32 (2.52 to 10.1)	< 0.01	6.19 (2.42–9.95)	< 0.01	3.02 (− 0.95 to 6.98)	0.14	–	–
Irregular	0.32 (− 0.52 to 1.16)	0.45	–	–	0.01 (− 0.89 to 0.91)	0.98	–	–
Posterior projection	0.15 (− 0.89 to 1.18)	0.78	–	–	0.44 (− 0.74 to 1.63)	0.46	–	–
Hypoplastic PCoA								
Unilateral (vs. no)	− 0.44 (− 1.60 to 0.71)	0.45	–	–	− 0.89 (− 2.04 to 0.25)	0.13	–	–
Bilateral (vs. no)	0.61 (− 0.52 to 1.74)	0.29	–	–	− 0.41 (− 1.62 to 0.80)	0.51	–	–
Aplastic PCoA								
Unilateral (vs. no)	− 15.6 (− 3002 to 2971)	0.99	–	–	− 3.49 (− 6.97 to − 0.01)	0.049	–	–
Bilateral (vs. no)	− 1.37 (− 3.8 to 1.12)	0.28	–	–	0.10 (− 2.43 to 2.62)	0.94	–	–
Fetal PCoA								
Unilateral (vs. no)	0.20 (− 1.78 to 2.17)	0.84	–	–	− 0.24 (− 2.13– to 0.64)	0.80	–	–
Bilateral (vs. no)**	–	–	–	–	–	–	–	–
Vertebral dominance	0.02 (− 0.98 to 1.02)	0.97	–	–	0.32 (− 0.79 to 1.43)	0.58	–	–
Maximum height in mm	0.05 (− 0.07 to 0.17)	0.40	–	–	0.02 (− 0.10 to 0.15)	0.72	–	–
Perpendicular height in mm	0.03 (− 0.08 to 0.15)	0.57	–	–	0.02 (− 0.11 to 0.15)	0.74	–	–
Diameter neck in mm	0.17 (− 0.42 to 0.23)	0.56	–	–	− 0.36 (− 0.71 to − 0.02)	0.04	–	–
Width aneurysm in mm	0.02 (− 0.12 to 0.15)	0.80	–	–	− 0.05 (− 0.20 to 0.09)	0.47	–	–
Height/width ratio	− 0.23 (− 1.41 to 0.95)	0.70	–	–	1.18 (− 0.64 to 3.00)	0.21	–	–
Daughter diameter ratio (larger/smaller)	2.62 (0.01–5.23)	0.049	–	–	1.60 (− 1.34 to 4.54)	0.29	–	–
Parent artery (basilar artery) diameter	− 0.35 (− 1.37 to 0.67)	0.50	–	–	− 0.24 (− 1.25 to 0.76)	0.64	–	–
Size ratio	0.25 (− 0.37 to 0.87)	0.43	–	–	0.05 (− 0.57 to 0.67)	0.88	–	–
Daughter-daughter angle	0.002 (− 0.02 to 0.02)	0.82	–	–	− 0.01 (− 0.03 to 0.02)	0.56	–	–

*71 patients with available Hunt-Hess scores and Fisher grades.

**No ruptured aneurysm had bilateral fetal PCoAs.

## Discussion

In this study, we showed that presence of a daughter dome, aspect ratio, and larger flow angle were significantly associated with basilar aneurysm rupture status. We also introduced two new robust parameters in this context, diameter size ratio and parent-daughter angle ratio, which were both significantly and inversely associated with ruptured basilar tip aneurysms. Of these parameters, the presence of daughter domes and aspect ratio are dependent on the aneurysm itself, while flow angle and parent-daughter angle ratio give the relationship between the aneurysm and the surrounding vasculature.

The association between multilobed aneurysms and rupture status has been shown before, and it is believed that multilobed aneurysms are to be in a more advanced stage of development with a greater risk of rupture^19–31^. We found a threefold increase in the association of multilobed aneurysms with rupture compared to non-multilobed aneurysms (63.0% vs. 19.7%), which is similar to previous reports^32,33^.

We also found diameter size ratio, one of the new parameters we introduced in this context, defined as the parent artery diameter divided by the sum of the diameters of both daughter branches, to be inversely associated with ruptured basilar tip aneurysms. Importantly, this parameter is unlikely to be changed by the formation of the aneurysm itself. Although we previously showed that an absolute smaller basilar artery diameter was significantly associated with aneurysm formation, we now provide a much more robust measure of the relative relationship between the diameter of the basilar artery and the daughter vessels^34^. Flow within the basilar bifurcation depends on a variety of geometric variables, including the relative caliber of the parent and daughter branches. It is believed that a smaller basilar artery diameter compared to the posterior cerebral arteries (e.g. a smaller diameter size ratio) provides a higher jet flow at the apex of the bifurcation, leading to a region of maximum hemodynamic stress, structural fatigue of the aneurysm wall, and consequent rupture^34–36^. Interestingly, we also found that higher diameter size ratio is associated with higher Hunt-Hess score, which is consistent with having a higher jet flow at the basilar apex.

Aspect ratio, which was calculated as the ratio of the maximum perpendicular height of the aneurysm to the average neck diameter of the aneurysm, was also significantly associated with ruptured basilar tip aneurysms. This finding is in line with Ambekar et al., which showed in a study of 31 ruptured and 17 unruptured basilar bifurcation aneurysms, that aneurysms with an aspect ratio of ≥ 1.9 were 6.3 times more likely to be ruptured than those with smaller aspect ratios. This association was also observed in middle cerebral artery aneurysms in other studies^9,24,37,38^.

We also showed that a larger flow angle (e.g. the angle between the maximum height of the aneurysm and the parent vessel) was significantly associated with aneurysm rupture. This finding was also observed by Ambekar et al. who showed that basilar bifurcation aneurysms that have their long axis in line with the basilar artery are more likely to be ruptured than those directed at an angle. It has been hypothesized that an increasing flow angle causes a higher inflow jet into the aneurysm, resulting in growth in the specific direction^39^. On the other hand, we found that smaller flow angle was associated with higher Fisher grade. This may be due to the effects of wall shear stress on platelet adhesion at the site of rupture, however further studies are required to elucidate this^40^.

Interestingly, we further found parent daughter angle ratio, another new variable we previously introduced for middle cerebral artery aneurysms^41^, to be inversely associated with ruptured basilar tip aneurysms suggesting that symmetry of daughter angles is associated with a higher risk of rupture. We hypothesize that increasing daughter branch asymmetry would be beneficial by converting a bifurcation aneurysm to sidewall configuration, leading flow to be diverted away from the aneurysm neck. Indeed, bifurcation aneurysms are thought to have a higher rupture risk than sidewall aneurysms, irrespective of location^42^. However, further research is needed to elucidate the exact mechanisms of aneurysm formation and rupture, which is a complex interaction of fluid dynamics, cellular biology, and structural mechanics.

The retrospective design is a main limitation of this study. Aneurysm rupture could have changed the aneurysm morphology, as suggested by Skodvin et al.^43^ Therefore, some of the associations found may be a result of rupture rather than causal risk factors. The association of rupture status with smaller basilar artery diameter (compared to daughter branches) may be the result of vasospasm due to rupture. Parameters were measured manually which may affect their accuracy. However, this would be more reflective of actual clinical practice. Assessment of these morphological variables in the clinical setting, possibly in combination with other imaging modalities such as vessel wall MRI, could contribute to the risk evaluation in these patients^44–48^. Finally, smoking status was obtained via medical records review and may not be completely accurate.

## Conclusion

We showed that presence of a daughter dome, aspect ratio, and larger flow angle were significantly associated with ruptured basilar tip aneurysms. In contrast, diameter size ratio and parent-daughter angle ratio were significantly inversely associated with rupture. Finally, we showed that vessel morphology, namely diameter size ratio, may influence the severity of the hemorrhage.

## Data Availability

The datasets generated during and/or analysed during the current study are available from the corresponding author on reasonable request.
